# Diagnosing the double burden of malnutrition using estimated deviation values in low- and lower-middle-income countries

**DOI:** 10.1371/journal.pone.0208525

**Published:** 2018-12-06

**Authors:** Midori Ishikawa, Tetsuji Yokoyama, Masaki Sagehashi, Naoki Kunugita, Hiroko Miura

**Affiliations:** 1 Department of Health Promotion, National Institute of Public Health, Saitama, Japan; 2 Department of Environmental Health, National Institute of Public Health, Saitama, Japan; 3 Department of International Health and Collaboration, National Institute of Public Health, Saitama, Japan; University of Dhaka, BANGLADESH

## Abstract

**Objective:**

To examine the possibility of diagnosing the double burden of malnutrition using estimated deviation values in low- and lower-middle-income countries.

**Methods:**

A modified version of the Japanese Diagnostic Tool was used. Data on 194 countries were analyzed, including data from the United Nations International Children’s Fund, World Health Organization and World Bank. After conducting a Box–Cox transformation, deviation values were calculated. The degree to which the values deviated relative to a deviation cutoff value of 50 was assessed. Focusing on countries with low- and middle-income economic levels, we examined the utility of this tool to show characteristic nutritional problems in each country.

**Results:**

The deviation values had normal, distorted, bimodal, or trimodal distributions. In the lower-middle-income countries, almost all countries had values ranging from 40 to 60 for education and water environments (urban and rural), and the differences were minimal. However, different causes of noncommunicable disease-related deaths were considered, and the primary cause appeared to be related to lifestyle factors, particularly alcohol consumption and tobacco smoking. In comparison, the deviation values related to death among low-income countries also appeared to be related to differences in education and sanitation in urban and rural areas.

**Conclusion:**

The study results can help to determine the status of nutritional inequalities and plan country-specific strategies to reduce the double burden of malnutrition.

## Introduction

The importance of monitoring diseases associated with under- and overnutrition at the country level and the identification of life-course factors that lead to noncommunicable diseases (NCDs) were recommended at the second International Conference on Nutrition (ICN2) [1–3].

In the “2030 Agenda for Sustainable Development” that includes the Sustainable Development Goals (SDGs), one of the main characteristics of the SDGs was that nutrition issues were clearly demonstrated in Goal two “Zero Hunger” and Goal three “Good Health and Well-being” [4]. A nutrition-related major risk factor for mortality is the reported lack of safe water, sanitation, and hygiene (WASH) services [5]. Globally, the stunting rate declined from 33% in 2000 to 23% in 2016, but Southern Asia and sub-Saharan Africa accounted for three quarters of all stunted children that year [4]. Because the economic situations of these countries are severe, it is important to consider ways to identify high priority issues related to nutrition for effective intervention. Additionally, monitoring of the indicators among nutrition-related holistic life-course viewpoints is necessary [6]. Human Development Reports in United Nations Development Program showed a comparable tool called the “The Multidimensional Poverty Index (MPI)” to analyze holistic viewpoints and to explore multidimensional approaches for eradication of poverty. In addition to explicit assessment of poverty, MPI utilizes numerous indicators to develop composite scores [7]. However, it does not focus on nutritional problems from life-course viewpoints. Analyzing the positioning of the problem among countries with similar economic situations may enable identification of clear nutritional tasks. However, there are very few reports on the types of methodology used to this effect.

Therefore, we attempted to apply diagnostic tools utilizing health-related data in Japan, which has recognized the importance of health disparities in its national plan “*Health Japan 21 (the second term)*” [8]. To prevent lifestyle-related diseases, it is necessary to clarify problems by identifying characteristics that are risk factors for certain health problems. As part of this larger plan, the “Data Health Plans” program has encouraged the analysis of health examination data, implementation and evaluation of health maintenance projects, and promotion of health insurance unions [9]. Consistent with these policies, local governments have attempted to analyze and monitor existing health data, including health examination data, medical prescriptions and national and local government (prefectural) survey results.

To analyze prefectural-level health situations, a deviation graph for medical and health examination data has proven to be informative. This tool expresses various health indicators as deviation values; thus, the strengths and weaknesses of the health index for specific prefectural/local governments can be examined [10]. The “Prefectural Diagnostic Tool” is currently only available in Japanese. It is important that “prefectural diagnostic tools” that are used in Japan be developed as tools that can be applied globally. This is because while comparing the strength and weaknesses of the health, nutritional, food, and environmental status of our country with those of other economically similar countries, it may be possible to grasp high priority concerns and clearly think about countermeasures.

The aim of this study was to investigate the global application of the deviation graph as a monitoring tool for diagnosing the double burden of malnutrition at the country level. Additionally, we focused on countries with low- and middle-income economic levels to assess the possibility of identifying characteristic nutritional problems in each country.

## Methods

### Study data

The Japanese Prefectural Diagnostic Tool [10] was modified to monitor various types of uniform, multi-sectorial variables; data on neonates, newborns, infants and adults (including pregnant mothers) were also included in the analysis.

Data on 194 countries were used for this study, including data from the United Nations International Children’s Fund (UNICEF) Annual Report 2014 on 53 nutrition-related variables covering the period from gestation to death [11], data from the WHO Global Status Report on NCDs [12], data on the population using at least basic drinking-water/sanitation services described in Water, Sanitation, and Hygiene for All (WASH) services in the Global Health Observatory Data Repository of the WHO [12,13] and World Bank [14,15] and information on the socioeconomic status of each country.

Socioeconomic variables included total population, gross national income (GNI) per capita, preprimary school participation (enrollment ratio and survival rate up to the last primary grade), secondary school participation (enrollment ratio), total adult literacy rate, and life expectancy at birth. Other variables were assessed across various life stages (gestational, fetal, newborn, infant and adult).

Indicators of undernutrition for preschool nutritional status included adjusted maternal mortality rate, low birthweight, neonatal mortality rate, infant mortality rate (aged <12 months), exclusive breastfeeding <6 months of age, introduction of solid food, adequate iodized salt consumption, vitamin A supplementation (full coverage), as well as the underweight, stunting, wasting, overweight, and mortality rates for children aged <5 years. Indicators related to undernutrition for WASH included diarrhea-related deaths due to inadequate WASH in children <5 years old, using at least basic drinking-water services (urban/rural/national), and using at least basic sanitation services (urban/rural/national). As defined, it could include using high quality water services [14,15].

Indicators of overnutrition included the prevalence of insufficient physical activity, consumption of alcohol per capita (liters of pure alcohol, crude-adjusted estimates), alcohol-related disorders (12-month prevalence), current tobacco smoking status; mean body mass index (BMI), overweight (BMI ≥25), obesity (BMI ≥30), raised blood pressure (systolic blood pressure ≥140 and/or diastolic blood pressure ≥90), raised blood glucose (fasting glucose ≥7.0 mmol/L, 126 mg/dl, medication for raised blood glucose or a history of diabetes diagnosis), and mortality rates per 100,000 for diabetes, cardiovascular disease, chronic respiratory diseases, cancers, and other NCDs. These variables were age-standardized unless otherwise stated.

### Calculating deviation values and data analysis

Taking into consideration that most variables were not normally distributed and many had a bimodal distribution even after a log transformation, a Box–Cox transformation was used for all variables [16]. Following the Box–Cox transformation, all variables were normally distributed. The deviation values were then calculated by using the following equation: deviation value = (number − average value) ÷ (standard deviation × 10) + 50. A deviation value of 50 was used as reference, and the extent (high or low) of deviation was evaluated [10].

If high average life expectancy and low mortality rate due to NCDs were the most desirable status, there were items for which higher values were desirable (e.g. school participation, total adult literacy rate, exclusive breastfeeding <6 months of age, etc.) and items for which lower values were desirable (e.g. stunting, overweight, current tobacco smoking status, etc.). Therefore, the deviation values of all items were modified so that a higher value would be desirable for easy and comprehensive analysis of the values of all the items.

A substantial proportion of the studied countries had nearly 100% primary school participation, literacy, and vitamin A supplementation rates. For these variables, the deviation values corresponded to their rank in the normal distribution. Specifically, each country’s percentile was represented by p (e.g., a country in the 25^th^ percentile was assigned p = 0.25), and this was used to determine the corresponding Z-scores. Next, a deviation value was calculated. The percentiles were also used to calculate the mean values for the variables. Finally, the deviation values of the variables for each life stage were presented graphically.

Deviation score is sometimes referred to as T-score, which is a standard score with a mean of 50 and a standard deviation of 10 [17]. The deviation scores can be interpreted as follows; for example, a country earning the score of 40 scored standard deviation below mean of 50, whereas a country earning the score of 60 scored standard deviation above the mean of 50.

The deviation values were grouped by economic income level classification [11]. Fifty-five countries had high-income levels, 57 had upper-middle levels, 48 had lower-middle-income levels and 34 had low-income levels. This study focused on countries with low- and lower-middle-income levels.

All statistical analyses were performed by using SAS software, version 9.2 (SAS Institute, Inc., Cary, NC, USA).

## Results

### Distribution of deviation values of indicators

The deviation values had normal, distorted, bimodal, or trimodal distributions. Normally distributed variables included total population; GNI per capita; life expectancy at birth; maternal mortality rate; exclusive breastfeeding <6 months of age; introduction of solid, semi-solid, or soft food at 6–8 months; adequate iodized salt consumption; prevalence of insufficient physical activity among men and women; per capita alcohol consumption; alcohol-use disorders among men; current tobacco smoking status among men; and mean BMI among women.

Variables with a distorted distribution included total adult literacy rate, vitamin A supplementation prevalence, prevalence of being underweight, prevalence of obesity among women, elevated blood pressure, elevated blood glucose among women and mortality rates for diabetes (men), cardiovascular disease (men), chronic respiratory diseases, and cancer.

Bimodally distributed variables were low birthweight, neonatal mortality rate, infant mortality rate, stunting, wasting, overweight, per capita alcohol consumption, mean BMI among men, obesity rate among men and mortality rates for diabetes (women), cardiovascular disease (women), and NCDs. The under-5-years-of-age mortality rate was trimodally distributed.

The numbers of countries varied depending on the indicators. Educational, WASH and tobacco indicators were fewer than others.

### Diagnosis of the deviation graph for lower-middle-income countries

**Fig 1:**
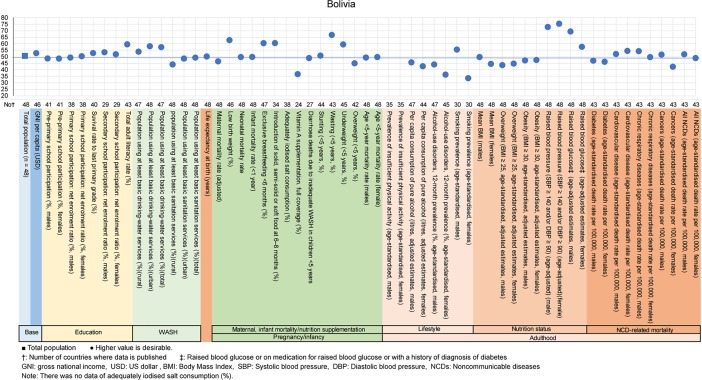
Country diagnosis by the deviation graph for Bolivia.

**Fig 2:**
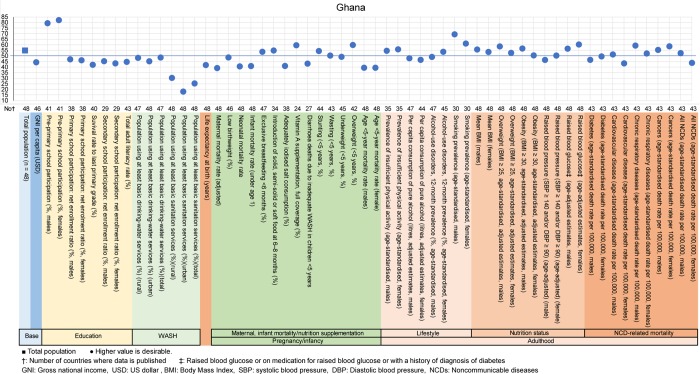
Country diagnosis by the deviation graph for Ghana.

**Fig 3:**
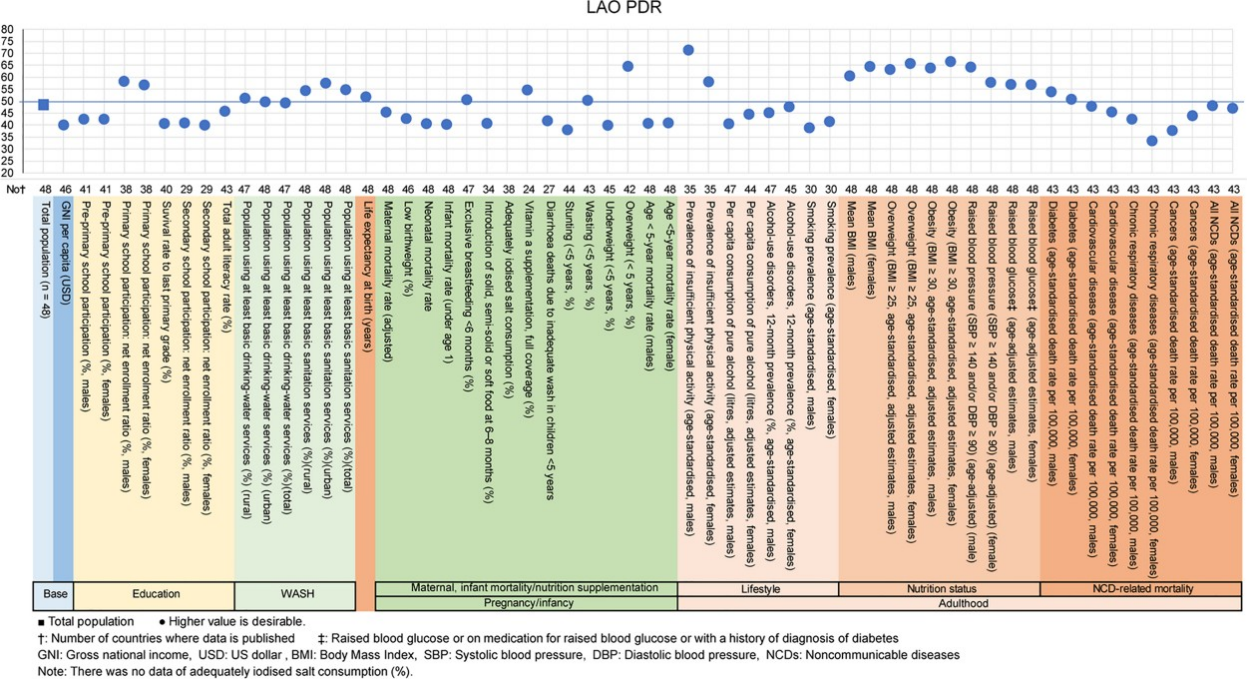
Country diagnosis by the deviation graph for Lao People's Democratic Republic (Lao PDR).

**Fig 4:**
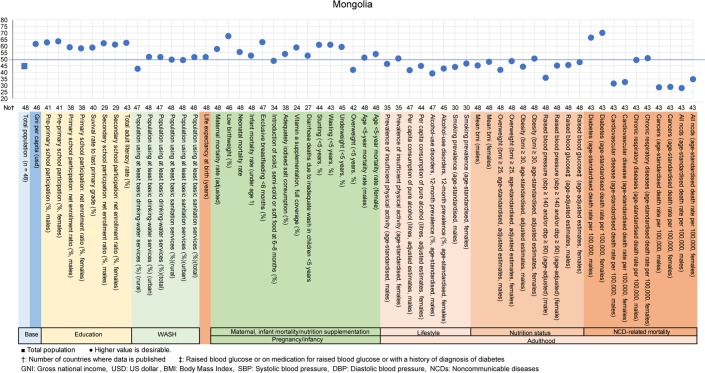
Country diagnosis by the deviation graph for Mongolia.

**Fig 5:**
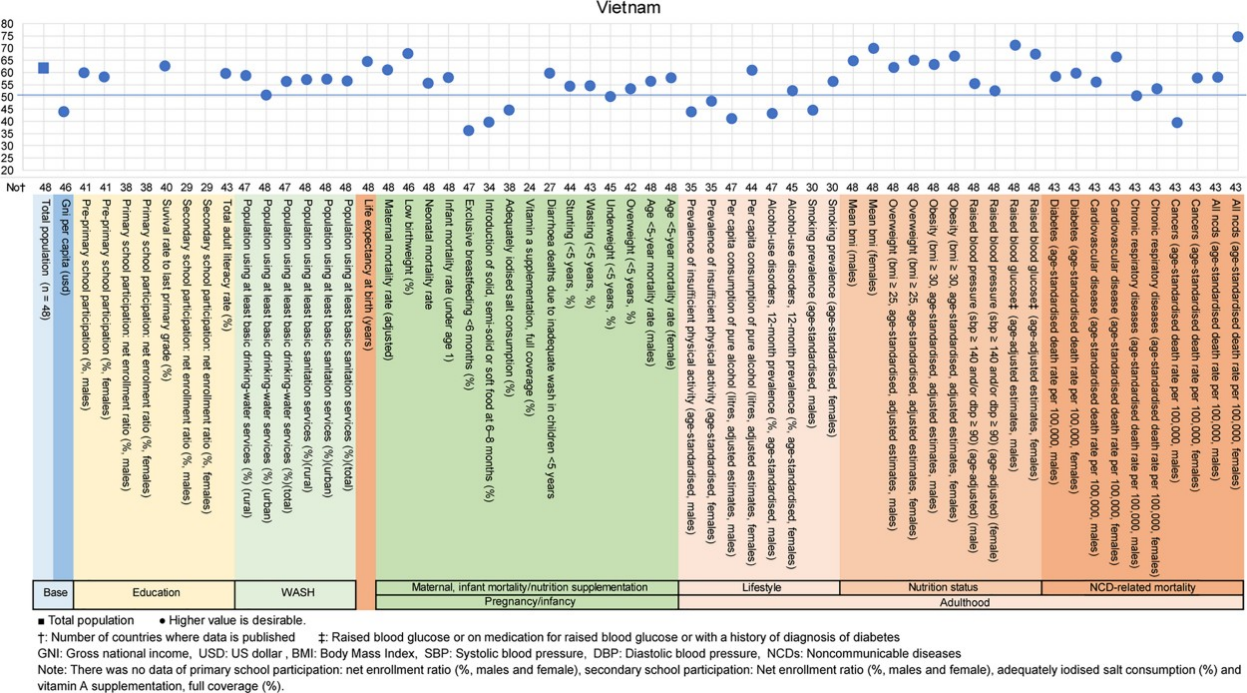
Country diagnosis by the deviation graph for Vietnam.

The examples of a country level diagnosis of the deviation graph for lower-middle-income countries are shown in Figs 1–5. Higher values were desirable except for the total population. The countries were Bolivia, Ghana, Lao People's Democratic Republic (Lao PDR), Mongolia and Vietnam. Five example countries of the lower-middle-income countries were chosen because these countries showed the data of all indicators, had the most significant results, and were also the most diverse.

In Bolivia, the deviation values were in the middle range at 53 for GNI per capita and 50 for life expectancy at birth and high at 60 for the total adult literacy rate. The deviation value for the exclusive breastfeeding rate was high at 60 and for being overweight at 5-years-of-age was low at 45.

The deviation values for rates of alcohol-use disorders and current tobacco smoking status for women were 36 and 34, respectively. The deviation values for increased blood pressure rates (SBP ≥140 and/or DBP ≥90) were very high for men and women at 73 and 75, respectively. The deviation values for the mortality rates for cancers were 42 for women and 51 for men.

In Ghana, the deviation values for the preprimary school participation rates were very high at 79 and 82 for men and women, respectively. However, the deviation values for the population using at least basic sanitation services were very low at 25. The value for life expectancy was low at 42, and the values for maternal, neonatal, infant, and <5 years old mortality rates were high at 39, 41, 41, and 40, respectively. Regarding NCD indicators, the deviation value for cardiovascular disease in women was low at 43.

In Lao PDR, the deviation values for primary school participation rates were high for men and women. However, the deviation values for low birthweight and neonatal mortality rates were low at 43 and 41, respectively. Conversely, the prevalences of physical activity and obesity rates were low for men and women.

In Mongolia, the deviation values for education rates were very high. However, the deviation values for the overweight rate for age <5 years was high at 58 and for cardiovascular disease and cancer rates were very high for men and women.

In Vietnam, the deviation value for life expectancy at birth was high at 64. The deviation values for alcohol consumption, alcohol-related disorder, tobacco smoking status, and cancer rates in men were low at 41, 43, 43, and 39, respectively.

All the above-mentioned countries had similar economic situations, but the characteristic nutritional problems were different. Some countries had problems in pregnancy and infancy, and some countries had problems with adult lifestyles.

### Diagnosis of the deviation graph for low-income countries

**Fig 6:**
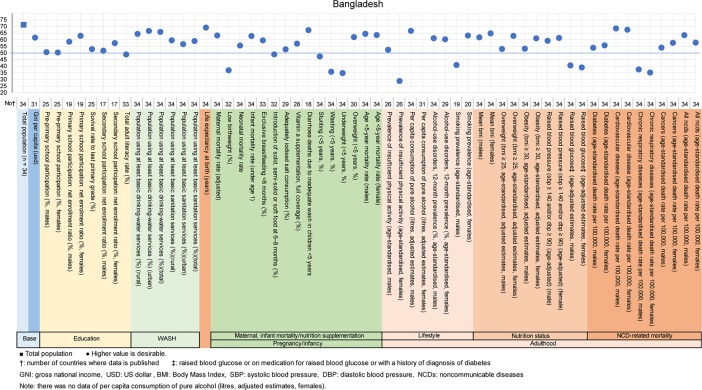
Country diagnosis by the deviation graph for Bangladesh.

**Fig 7:**
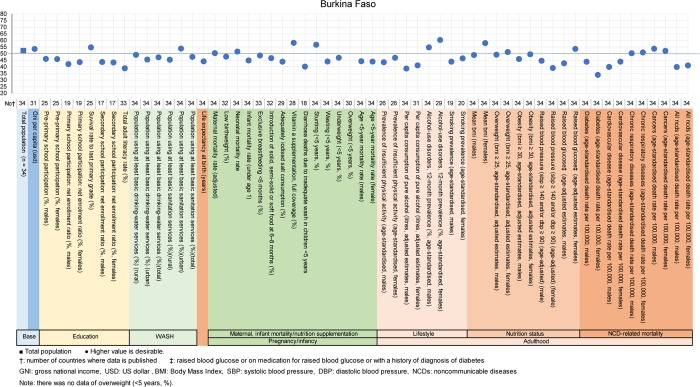
Country diagnosis by the deviation graph for Burkina Faso.

**Fig 8:**
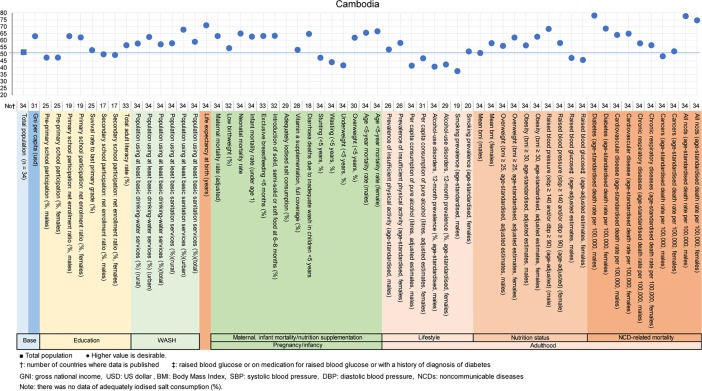
Country diagnosis by the deviation graph for Cambodia.

**Fig 9:**
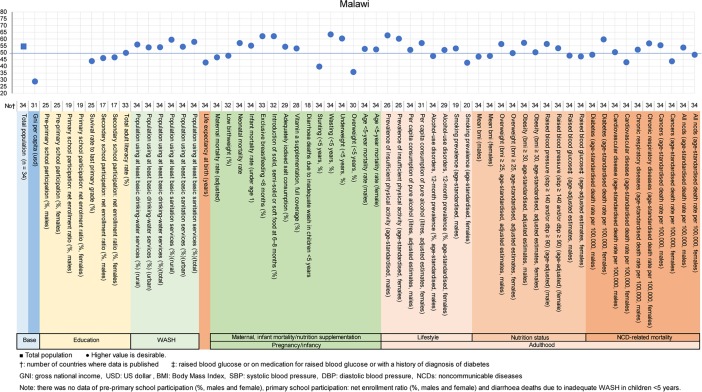
Country diagnosis by the deviation graph for Malawi.

**Fig 10:**
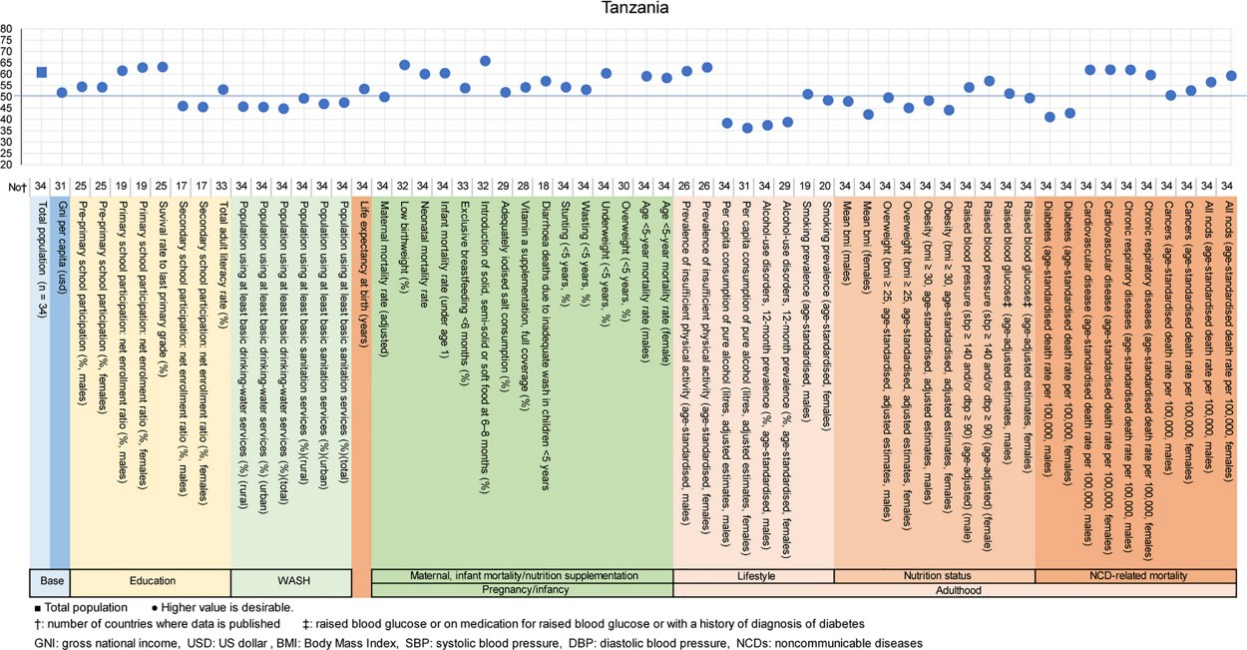
Country diagnosis by the deviation graph for Tanzania.

Examples of country level diagnoses for low-income countries are shown in Figs 6–10. The countries presented are Bangladesh, Burkina Faso, Cambodia, Malawi, and Tanzania. Five example countries of the low-income countries were chosen because these countries showed the data of all indicators, had the most significant results, and were also the most diverse.

In Bangladesh, the deviation values were high at 62 for GNI per capita, 69 for life expectancy at birth, and were high for education and WASH indicators. On the other hand, the deviation value for low birthweight was low at 37 relatives to the deviation value of 63 for the maternal mortality rate. The deviation values for physical activity in women (29) and smoking prevalence in men (41) were low, which is not desirable. The deviation values of raised blood glucose and chronic respiratory diseases were low in men and women.

In Burkina Faso, the deviation values for total adult literacy rate and diarrhea-related deaths due to inadequate WASH in children <5 years old were low at 39 and 40, respectively. Additionally, the deviations values for raised blood pressure in women and diabetes in women were low.

In Cambodia, the deviation values for the population using at least basic drinking-water, sanitation services were high. Similarly, the deviation value for life expectancy at birth was high at 71 also. In Cambodia, the major issues were alcohol consumption and tobacco smoking.

In Malawi, the deviation values for stunting and being overweight at <5 years old were low at 40 and 36, respectively. The deviation values for cardiovascular disease and cancer rates in women were also low at 43 and 44.

In Tanzania, the deviation values for alcohol consumption, obesity rate, and diabetes in men and women were low.

All the above-mentioned countries had similar economic situations, but the characteristic nutritional problems were different. There did not appear to be any country with nutritional problems during both the infant and adult age periods.

## Discussion

The results of deviation value analysis among low-income or lower-middle-income countries showed that the more important nutritional issues varied by country, even among those with similar economic situations. In the lower-middle-income countries, the deviation values for most countries ranged from 40 to 60 for education and water environments (including urban and rural), and the differences were not large. However, different causes of NCD-related death were considered, and the primary cause of death appeared to be related to lifestyle, especially alcohol consumption and tobacco smoking.

Comparatively, the deviation values related to death among low-income countries appeared to be related to differences in preprimary, primary and secondary schools education [18] and in sanitation in urban or rural environments [19,20]. In addition, nutritional differences were observed in early childhood during the newborn period among countries. These nutritional differences during the newborn and early childhood periods could potentially affect later health [21,22].

Although high-income countries were not aimed in the present study, results from previous research can be useful in considering the possible application of deviation graph research to those countries. Among high-income countries, the deviation values for high blood pressure and cancer-related mortality have been previously reported to be lower in Japan. Those deviation values have been shared among health staff and used to prioritize projects. Deviation graph results have also been previously used to provide guidance when promoting collaboration between departments on data analysis, and therefore, the use of deviation graph research could potentially lead to increased cooperation between health centers and hospitals [9].

We found the deviation graph tool used in this study to have the following characteristics. First, we proposed using graphs of estimated deviation values, which is an approach currently used at the prefecture level in Japan. This tool for analyzing local health inequalities can be potentially applied globally as a monitoring tool for diagnosing the double burden of malnutrition at the national level. This could be accomplished by using existing information published by UNICEF, WHO, and the World Bank that is related to under- and overnutrition-related chronic diseases. We suggest that analyzing the strengths and weaknesses of the health indexes of different countries is possible. Consequently, this analysis can help to both determine the status of nutritional inequalities and plan country-specific strategies for reducing malnutrition. Applying such an approach to diagnose malnutrition worldwide can contribute to building agreements in terms of priority areas for intervention and development.

However, this approach may be limited because of variations in reporting among countries [11,12].

Only few countries have reported vitamin A supplementation and diarrhea-related deaths due to inadequate WASH in children <5 years of age, which makes it difficult to calculate deviation values for these variables. Because the deviation score indicates the relative position of a country among the countries included in the analysis, missing data of some countries could affect the scores of other countries. Therefore, deviation scores among various variables in a country should be compared while considering the included countries. The Box–Cox transformation is the best approach for dealing with this data and permits long-term monitoring of trends.

Second, applying the described approach and using existing data published by other organizations may facilitate the monitoring of SDGs.

This method facilitates identification of data types that are not reported at the country level [23]. Third, to ensure the high quality of data reported in each country, cooperation between international organizations is important.

Despite these limitations, we could have explored some of these issues with the current data. The study would be valuable to countries that may want to analyze the situations in their countries using a global monitoring tool.

Prior research has observed that a standardized tool for monitoring malnutrition that can be used globally is unavailable. The present study results indicate that analysis of the strengths and weaknesses of the health indexes of different countries is possible [6]. Consequently, this analysis can help to both determine the status of nutritional inequalities and plan country-specific strategies for reducing the double burden of malnutrition. Further, in discussing the SDGs, all countries highlighted the importance of confirming their positions on global issues [24]. The analyses presented here may contribute useful information to all countries participating in the global effort to reach the SDGs [25,26].

## Supporting information

S1 File (DOCX)
