# Supplementary material for: Diagnosing the double burden of malnutrition using estimated deviation values in low- and lower-middle-income countries
Source: PLoS One. 2018 Dec 6;13(12):e0208525. doi: 10.1371/journal.pone.0208525 (PMC6283532; doi:10.1371/journal.pone.0208525)
Supplement: S1 File — (DOCX) [file pone.0208525.s001.docx]

**Data availability statement**

"1. UNICEF. Annual report 2014 [Internet]. New York: UNICEF; 2014 [cited 2018 November 7]. Available from:
https://www.unicef.org/publications/index_82455.html
2. WHO. Global status report on noncommunicable disease 2014 [Internet]. Geneva: WHO; 2014
[cited 2018 November 7]. Available from: http://www.who.int/nmh/publications/ncd-status-report-2014/en/.
3. WHO SDG data. Global Health Observatory resources. [cited 2018 November 7]. Available from
http://apps.who.int/gho/data/node.resources
4. World Bank Group. Country data tables; IBNET indicator. The IBNET Water supply and sanitation Blue Book
2014. Washington DC; 2014. p. 37-139. [cited 2018 November 7]. Available from:
Response to Reviewers
https://openknowledge.worldbank.org/handle/10986/2545
5. Global status report on water safety plans [cited 2018 November 7]. Available from:
http://apps.who.int/iris/bitstream/handle/10665/255649/WHO-FWC-WSH-17.03-
eng.pdf;jsessionid=48EC9A5C12B725031E6F3382487BAE9A?sequence=1"
